# GOLD or lower limit of normal definition? a letter and authors’ response

**DOI:** 10.1186/1465-9921-13-61

**Published:** 2012-07-26

**Authors:** Philip Quanjer, Carlos A Vaz Fragoso, Martin R Miller, Gülmisal Güder, Stefan Störk, Arno W Hoes, Frans Rutten

**Affiliations:** 1 Department of Pulmonary Diseases and Department of Paediatrics, Erasmus Medical Centre, Erasmus University, Rotterdam, the Netherlands; 2 Department of Internal Medicine, New Haven, CT, USA, and Veterans Affairs (VA) Clinical Epidemiology Research Center, Yale University School of Medicine, West Haven, CT, USA; 3 Institute of Occupational and Environmental Medicine, University of Birmingham, Birmingham, UK; 4 Julius Center for Health Sciences and Primary Care, University Medical Center Utrecht, Utrecht, The Netherlands; 5 Comprehensive Heart Failure Center, Department of Internal Medicine I, University Würzburg, Würzburg, Germany

## Letter

Philip H Quanjer, Carlos A Vaz Fragoso and Martin R Miller.

It is a very refreshing idea to use clinical data and health outcomes to decide how to best diagnose Chronic Obstructive Pulmonary Disease (COPD). Therefore we read the article by Güder *et al*. [1] with great interest. However, upon reading the article in more detail we developed reservations about the criteria used.

The authors started with 405 patients aged ≥65 years with a general practitioner’s (GP) diagnosis of COPD. A GP and a chest physician subsequently decided that 37% did not have COPD but they were still treated as if they had COPD. In fact, the percentage of not-COPD subjects receiving inhaled corticosteroids was almost as high as in those with expert panel (EP) COPD (EP COPD+). As there was a definite COPD bias in the selection of subjects, the EP-COPD negative patients are not an adequate control group.

A large mix of criteria was used to diagnose EP-COPD+. It is impossible to reconstruct how the diagnosis was adjudicated. There does not seem to have been any weighting of symptoms, signs, or measurement results. In fact, FEV_1_/FVC, FEV_1_ percentage of predicted, RV/TLC were used, but no information is provided what criteria were applied in diagnosing EP-COPD+. Presumably the GP and chest physician adjudicated a diagnosis without a predefined algorithm, nor blinded to the assessment of their counterpart. It is to be expected that the judgment of the chest physician would predominate, because many of the measurements are not part and parcel of a GP’s routine. In that respect the fall in Cohen’s kappa upon re-assessing the EP-COPD diagnosis suggests that agreement between chest physicians is not optimal.

The diagnosis of COPD was based on pulmonary function indices, using unspecified cutoff criteria and an unknown reference equation. Pulmonary function criteria were probably pivotal in establishing the diagnosis. Maintaining a diagnosis based on arbitrary lung function criteria rather than redefining EP-COPD + by applying other pulmonary function criteria and prediction equations is a flawed approach, particularly when studying how the diagnosis of COPD is affected by such criteria and prediction equations. The exclusive use of one set of arbitrary pulmonary function criteria for establishing a diagnosis precludes studying how the diagnosis is affected by other criteria.

The use of percent of predicted, for example for FEV_1_, is also flawed as the scatter is not proportional to the predicted value; this creates an age, sex, height and ethnic group related bias [2-6]. Stanojevic [6] was the first to show that in adults the scatter increases with age. This means that the lower limit of normal (LLN) derived from conventional regression equations cannot be derived from predicted minus 1.64RSD, because the RSD varies with age. The use of z-scores using Stanojevic equations [6] is the method of choice, because z-scores are free of bias. The only problem is that the age is limited to 80 years; this will be resolved when the Global Lungs Initiative publishes its 3-95 year predicted values (http://www.lungfunction.org).

The number of co-morbidities in elderly subjects is very great and this makes it very difficult to tease out a clinical diagnosis of COPD. Cox regression (hazard ratios) should not be used as a basis for comparisons of risk among the various definitions of COPD, because these differ in their reference groups and because the expert panel would have had access to relevant information concerning likelihood of the tested outcomes. Moreover, the analyses make no adjustment for confounders such as age, smoking habit and male sex which will affect the tested outcomes and may vary between the groups.

Finally, including criteria to which a GP does not have access, such as RV/TLC, makes establishing a diagnosis of COPD out of reach of GPs.

## Authors’ response

Gülmisal Güder, Stefan Störk, Arno W Hoes and Frans H Rutten.

We thank Quanjer and colleagues for their insightful comments regarding our article [1]. Some points, however, deserve further clarification. The critique of current COPD definitions was not intended to specifically address the GOLD guidelines or the lower limit of normal (LLN) approach, but was a general caveat to base the diagnosis onto one single ratio derived from pulmonary function testing (PFT), ie FEV/FVC. Using the Stanojevic equation [6] as proposed by Quanjer might indeed increase the specificity of the LLN definition, but it will not improve the sensitivity since the LLN approach as well as the GOLD definition fails to acknowledge false negative spirometric test results. Important diagnostic needs will still remain unsolved. E.g., how should we classify patients who – according to the GOLD or LLN definition – are (borderline) normal and have abnormalities at diffusion testing or RV/TLC ratio, especially those with a smoking history and symptoms.

All 405 patients in our study had a GP’s diagnosis of COPD and were treated accordingly. The study team did not attempt to modify the pulmonary treatment. We also did not exclude patients with co-morbidities as this might provoke selection bias, nor did we consider healthy never-smokers as controls, since they hardly pose diagnostic problems in daily routine. According to the GOLD definition, about 60% of all patients suffered from COPD, and about 40% depending on the type of LLN definition. Hence, the challenge was not only to differentiate true positive from false positive COPD patients but to also consider the possibility of false negatives. We therefore used an expert diagnosis of a pulmonologist and GP (validated internally and externally) as the reference standard. We agree that this is not a perfect standard. However, we are still convinced that an experienced physician will outperform a single and rather rigid mathematical model in the complex process of integrating very heterogeneous informations from various tests, including other PFT results as well as signs and symptoms. For many diseases that lack an irrefutable single test as a reference, the use of an expert panel (using all diagnostic information) is an excepted alternative, e.g. in heart failure.

Furthermore, although the LLN approach is regarded more “physiological” than the GOLD definition, since it considers age-, sex-and height-dependent cut-off values of FEV_1_/FVC, the present real-world cohort illustrates that the LLN definition is not widely used by GPs to diagnose COPD. One reason for this reluctance might be the lack of consistency between different LLN equations [7]. In a recent review, the shortcomings of population-derived LLN equations were thoroughly discussed and the need of more studies with longitudinal data to identify markers of COPD diagnosis and prognosis was emphasized [8].

In our prospective cohort study the two most important PFT parameters for identifying a positive expert COPD diagnosis besides FEV_1_/FVC ratio were FEV_1_ as % predicted and RV/TLC. Our proposed diagnostic algorithm is not yet validated; however, it is the very first mathematical attempt that considers plausibility checking of a COPD diagnosis.

We agree with Quanjer and colleagues that in our Cox regression analysis comparing expert COPD vs. the LLN or GOLD definition information bias may be a concern and it may explain the slightly superior point estimate of the expert diagnosis. However, since cut-off based definitions do not consider other information than provided in the equation, multivariable adjustment is not justified to test the prognostic capacity of different LLN definitions as suggested by the authors. Instead, and consistent with general clinical practice, we advocate to deliberately use otherwise neglected clinical information for diagnosis and decision making.

In general, the goal of making a diagnosis is to define a certain population that may benefit from certain therapies. The ideal diagnostic perfectly identifies the subgroup sensitive to therapy, and accurately predicts relevant clinical outcomes (e.g., exacerbations, hospitalizations, mortality). Our results remind us of the usefulness of the clinical view, including signs and symptoms. Moreover, we provide some evidence that bodyplethysmographic measurements and diffusion testing is useful to decide whether a patient has COPD when spirometry results are indifferent. Maybe it is time to realize that the definition of COPD needs to include some ‘clinical items’, as nearly all definitions of diseases in medicine include clinical aspects of patients.
